# Nitrifier abundance and diversity peak at deep redox transition zones

**DOI:** 10.1038/s41598-019-44585-6

**Published:** 2019-06-14

**Authors:** Rui Zhao, Bjarte Hannisdal, Josè M. Mogollon, Steffen L. Jørgensen

**Affiliations:** 1 Department of Biology, University of Bergen, Thormøhlens gate 53A, Bergen, 5007 Norway; 2 K.G. Jebsen Centre for Deep Sea Research, Department of Earth Science, University of Bergen, Allegaten 41, Bergen, 5007 Norway; 3 Institute of Environmental Sciences (CML), Leiden University, Leiden, Netherlands; 4 Present Address: School of Marine Science and Policy, University of Delaware, Lewes, DE 19958 USA

**Keywords:** Marine biology, Element cycles

## Abstract

More than half of the global ocean floor is draped by nutrient-starved sediments characterized by deep oxygen penetration and a prevalence of oxidized nitrogen. Despite low energy availability, this habitat hosts a vast microbial population, and geochemical characteristics suggest that nitrogen compounds are an energy source critical to sustaining this biomass. However, metabolic rates of nitrogen transformation and their link to microbial survival in this global-scale ecosystem remain virtually unknown. Here we provide quantitative constraints on microbial nitrogen cycling in open ocean oligotrophic sediments from seafloor to basement, spanning approximately 8 million years. We find active microbial nitrogen transformation throughout the sediment column but at very low rates. Local peaks in diversity and abundance of nitrifiers and denitrifiers occur at redox transition zones deep within the sediments, strongly indicating that these microbes are revived from their maintenance state and start growing again after millions of years of attrition.

## Introduction

Microbial communities in marine sediments impact all major global biogeochemical cycles, including those of oxygen, carbon, nitrogen, manganese, iron, and sulfur^1–4^. By mediating the flux of chemical compounds in and out of the sediment, this hidden biosphere influences the composition of the ocean and the atmosphere. Many of the processes constituting the global element cycles can occur without microbial catalyzation, albeit often at a considerably lower rate; the nitrogen cycle, however, is unique in that it is almost exclusively dependent on redox reactions regulated by microorganisms^5^. Hence, not only are the microbes essential for keeping the cycle turning^6^, but also for making nitrogen bioavailable and thereby a key controlling factor on primary production.

It is clear that microorganisms in marine sediments play a substantial part in balancing the global nitrogen cycle^6^, but despite recent progress in our understanding of nitrogen transformations in the marine environment^6–11^, our knowledge of key processes, fluxes, turnover rates, energy yields, spatial distributions, and population sizes in deep-sea sediments is strikingly limited^12^. One major reason for this limitation is the historical focus on organic-rich sediments on continental margins or in upwelling zones^13^. In such systems, the availability of energy, in the form of organic carbon, is accompanied by high microbial activity, restricting oxygen and nitrate penetration to a depth of centimeters or less^14,15^. Hence, the importance of nitrogen transformation in sediments with high organic load is dwarfed by other redox reactions such as sulfate reduction, for which the reaction zones can extend to depths of several hundred meters^2,4^. However, as researchers turn their attention to the vast oligotrophic regions on the ocean floor, it is becoming apparent that deep oxygen penetration and persistent nitrate throughout the sediment column represent a widespread geochemical scenario that differs markedly from the classic diagenetic redox sequence of organic-rich sediments^16–21^. Considering that more than half of the ocean floor is draped by oligotrophic sediments^22^, these new insights suggest that nitrogen transformation is a pervasive process in the deep sedimentary biosphere.

We present a comprehensive investigation of microbial nitrogen cycling in oligotrophic deep subseafloor sediments, using samples collected from North Pond, a small sediment filled basin, on the western flank of the Mid-Atlantic Ridge visited during the Integrated Ocean Drilling Program (IODP) Expedition 336. Two sediment cores from Holes U1383E and U1384A, hereafter referred to as site 3E and site 4A, respectively, provide access to the entire sediment column from seafloor to basement^23^. North Pond sediments are characterized as oligotrophic, with a total organic carbon content of <0.3 weight percent^16,24^. With deep oxygen penetration and nitrate present throughout the sediment^19,25^, these cores offer an ideal opportunity to study nitrogen transformation processes in deep oligotrophic subseafloor sediments. Our approach is to 1) study the nature and long term fate of the nitrogen transforming population as a function of depth, by characterizing the diversity and structure within this part of the community. 2) Combine *in situ* reaction rates predicted by reaction-transport models with quantitative microbial abundance data to derive the mean cell-specific metabolic rates of functional groups involved in nitrogen cycling. We find a predominance of nitrifiers and denitrifiers surviving in a maintenance state, with strong indication of revival and local growth driven by increased energy supply in transition zones between oxic and anoxic regimes deep within these oligotrophic sediments.

## Results

### Geochemical profiles and fluxes

**Figure 1:**
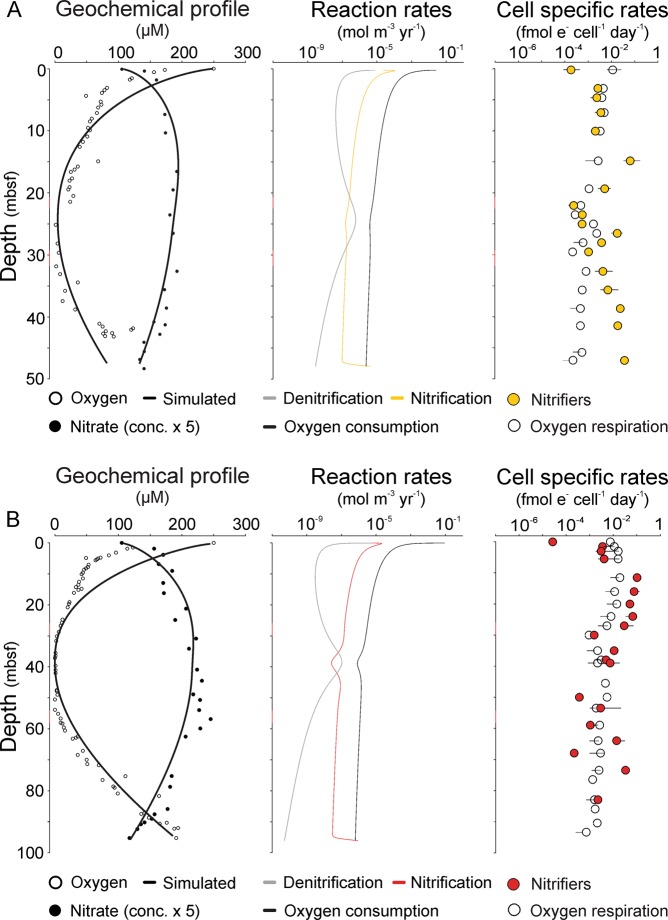
Geochemical profiles and reaction rates. Sediment core 3E (**A**) and 4A (**B**) displays Left to Right: (1) Nitrate and oxygen profiles. circles represent observed concentrations and solid lines simulated model output. Note that nitrate concentrations are upscaled with a factor five relative to true values. (2) Calculated reaction rates for nitrification (yellow and red lines in 3E and 4A, respectively), denitrification (grey lines) and oxygen consumption (black stipulated lines). (3) Cell specific reaction rates estimated from bulk volumetric reaction rates divided by functional gene abundances for nitrification (yellow and red circles in 3E and 4A, respectively) and bulk oxygen respiration (open circles). Error bars indicate standard deviation from qPCR quantification of functional genes.

At each site, the dissolved oxygen depth profile shows a characteristic “C” shape, with high concentrations at both the sediment-water interface and sediment-basalt interface, and decreasing concentration towards a central anoxic zone^25^ (Fig. 1). This pattern is partly caused by diffusion of dissolved oxygen into the sediment from the overlying seawater and from the underlying oxic basaltic aquifer, resulting in two distinct redox transition zones at each site: an oxic-anoxic transition zone (OATZ) and an anoxic-oxic transition zone (AOTZ) (Fig. 1). Following the traditional oceanographic definition, we identify the OATZ as an interval over which the O_2_ concentration drops below 10 µM down to the detection limit of ≤3 μM^26^ and, correspondingly, we define the AOTZ as the interval over which the concentration increases above detection limit and up to 10 µM. Consequently, the OATZs is found 21–24 meters below seafloor (mbsf) at site 3E and 26–30 mbsf at site 4A, while the AOTZs is located at 28–33 mbsf at 3E and 54–58 mbsf at 4A, respectively (Fig. 1, Table S1). The integrated O_2_ consumption in the OATZ (i.e. the difference between O_2_ fluxes in and out of the zone) accounts for 4% of total O_2_ influx from seawater at 4A (low data resolution prevented estimates for the OATZ at 3E), while the AOTZs account for 29% and 44% of O_2_ influx from the underlying basement at 4A and 3E, respectively (Table S1).

Nitrate concentrations in the pore water are higher than values measured in the bottom seawater and in the crustal fluids [~21.1 µM^16,27^] throughout both sediment cores^19^. Depth profiles of nitrate are mirror images of the oxygen pattern at both sites, with nitrate increasing downcore all the way into the AOTZ, reaching a maximum of 40–50 µM (Fig. 1). Below these maxima, nitrate concentrations decline and approach the bottom seawater values at the sediment-basalt interface. High sedimentary nitrate concentrations result in an efflux of nitrate from the sediment into both the overlying seawater (0.031–0.047 mmol m^−2^ yr^−1^) and the underlying oceanic crust (0.010–0.011 mmol m^−2^ yr^−1^), with the latter accounting for 19–24% of total sedimentary nitrate efflux (Table S1). Pore water nitrite and manganese concentrations were below the detection limit throughout both cores. The same was true for ammonium at site 4A, while low values ranging between 5–25 µM were found at site 3E^19^ (Supplementary Fig. S6).

### Quantification of functional genes and microbial population size

**Figure 2:**
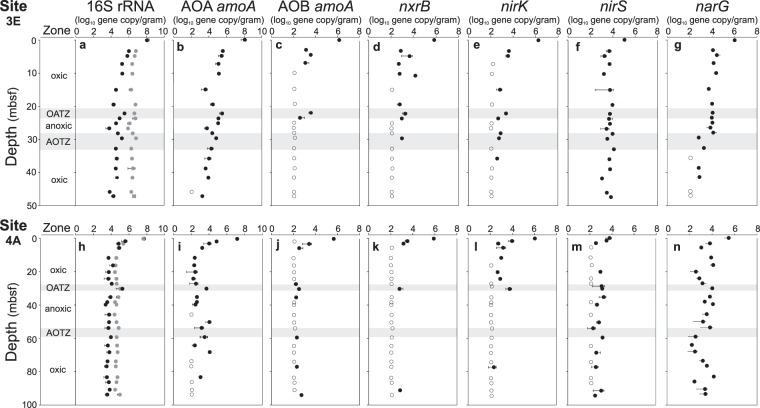
Functional gene abundances. Quantified gene copy numbers by qPCR in sediment cores 3E (panel a–g) and 4A (panel h–n). Left to Right: Oxygen regime, 16S rRNA (Archaea black circles and Bacteria grey circles), archaeal amoA, bacterial amoA, nxrB, nirK, nirS and narG. Open circles indicate abundances at or below detection limit (102 copies g-1 sediment). The oxic/anoxic transition zones (OATZ) and the anoxic/oxic transition zones (AOTZ) are marked with grey-shaded area.

At both sites, the estimated total abundance of microbes (the sum of archaeal and bacterial 16S rRNA genes) was highest near the surface, with ~10^8^ copies g^−1^ wet sediment, and then decreased with depth to a relatively stable level of ~10^5^–10^6^ at site 3E and ~10^4^–10^5^ at site 4A (Fig. 2). However, distinct abundance peaks were observed for both Bacteria and Archaea in the OATZs at both sites and less pronounced, in the AOTZ at 3E (Fig. 2).

The abundance of functional groups involved in nitrogen transformation was quantified by targeting their diagnostic functional genes (Fig. 2). Out of 12 target genes, we successfully detected six genes that encode key enzymes involved in nitrification and denitrification processes (Fig. 2, Table S4 show a complete list of targeted genes). In the uppermost sediments, the functional gene abundances are similar across cores, but decrease more rapidly with depth at 4A, resulting in lower overall abundances at this site (Fig. 2). Ammonia-oxidizing archaea (AOA), detected via the archaeal *amoA* gene, account for almost the entire archaeal population in the surface sediments, but decrease sharply with depth by four to five orders of magnitude. However, we found local archaeal *amoA* increases of up to 100 fold in the OATZ and AOTZ at both sites (Fig. 2). Ammonia-oxidizing bacteria, AOB, detected via the bacterial *amoA* gene, occur primarily in the uppermost six meters and in the OATZs, with abundances approximately two orders of magnitude lower than archaeal *amoA* throughout both cores (Fig. 2). Nitrite-oxidizing bacteria (NOB), which catalyze the second step in the nitrification process (oxidizing nitrite to nitrate), were detected via the *nxrB* gene, with a vertical distribution and abundance similar to that of the bacterial *amoA* (Fig. 2), including the local abundance increases in the OATZs at both sites. Additionally, we successfully detected genes encoding nitrate reductase (*narG*) and nitrite reductase (*nirK* and *nirS*), which are key enzymes that catalyze the first two steps of denitrification, respectively (NO_3_^−^ → NO_2_^−^ → NO). The abundance of the *narG* gene in 3E shows an initial decrease of two orders of magnitude, after which it stabilizes around 10^4^ copies per gram sediment from five mbsf and until the AOTZ where a further decline is observed (Fig. 2a). The *narG* distribution in 4A is more variable with apparent elevations at or near the two redox transition zones (Fig. 2b). The nitrite-reductase gene *nirK* is generally more abundant than *nirS* in the uppermost sediments, but has a more pronounced decrease with depth (Fig. 2), resulting in *nirS-*dominated denitrifier communities in deeper sediments. Slight increases of *nirK* occur in the OATZs, whereas *nirS* gene copy numbers are more evenly distributed (Fig. 2). The following functional genes were not detectable by qPCR in any of the analyzed samples: anammox bacteria (*hzsA* and *hzo* genes), DNRA bacteria (*nrfA* genes) and nitrogen-fixing bacteria (*nifH* genes), suggesting that these microbes play a negligible role in nitrogen cycling at the investigated depths.

### Prokaryotic community composition

**Figure 3:**
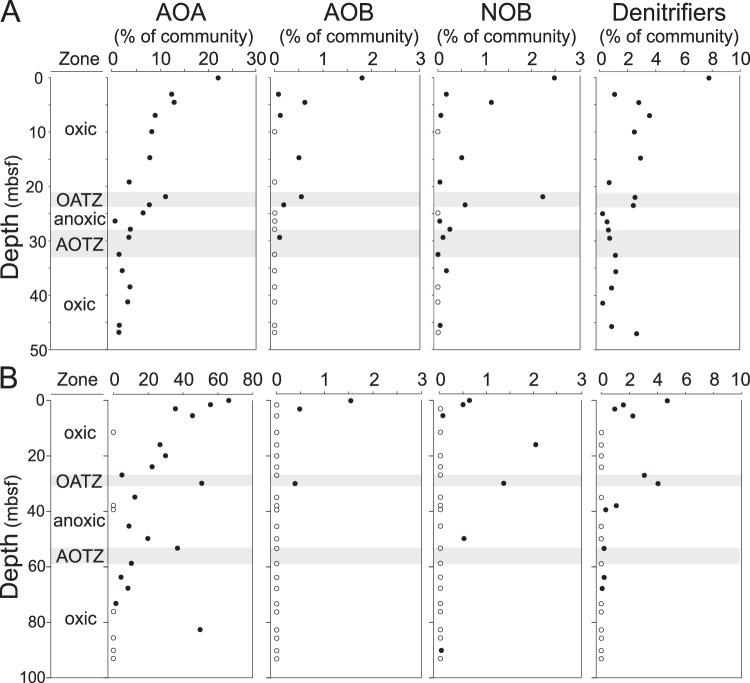
Functional group abundances. Values are based on taxonomic affiliation of partial 16S rRNA genes in core 3E (**A**) and 4A (**B**) and given as percent relative abundances of the total community population. Left to Right: Oxygen characteristics, putative ammonium oxidizing archaea (AOA), ammonium oxidizing bacteria [AOB], nitrite oxidizing bacteria (NOB) and denitrifiers (see material and methods for taxa included in each functional group). The oxic/anoxic transition zones (OATZ) and the anoxic/oxic transition zones (AOTZ) are marked with grey shaded area. Note the difference in range of x-axis values of AOA between the two cores.

A substantial fraction of the total community are putative nitrifiers (AOA, AOB, and NOB) and denitrifiers, based on the 16S rRNA gene amplicons, and their vertical distribution is largely consistent with the abundance variation of the corresponding functional genes (Figs 2 and 3). Putative AOA belonging to the order Nitrosopumilales [formerly known as Marine Group I/MG-I/marine group 1.1a^28^], within the phylum Thaumarchaeota occur in most samples and account for 21% and 66% of total richness in near-surface sediments at 3E and 4A, respectively (Fig. 3A,B). Their relative abundance decreases downcore but distinct peaks occur at the OATZs at both sites and in the AOTZ at site 4A. Occurrences of the AOB genera *Nitrosospira* and *Nitrosococcus* (Figs 3 and S5a) are consistent with the distribution of the bacterial *amoA* gene detected by qPCR (Fig. 2). Putative NOB affiliated with the genera *Nitrospina* and *Nitrospira* are also detected at most depths (Figs 3, S5b). Although the relative abundances of AOB and NOB (<3% of the total community) are much lower than AOA, their vertical distributions display similar distinct peaks in the OATZs (Fig. 3). Potential denitrifiers are also detected in our amplicon sequences and include members from *Aeromonas*, *Arcobacter, Pseudomonas* and *Woeseiaceae* [formerly known as JTB255-MBG^29^], all of which show elevated abundances in the OATZs (Fig. 3, and Tables S5 and S6). However, we note that functional prediction based solely on partial 16S rRNA gene information is challenging, especially of denitrifiers due to their high phylogenetic and metabolic diversity. Consistent with the qPCR results, we found little or no evidence for taxa suspected to be involved in anammox, DNRA, or nitrogen fixation.

**Figure 4:**
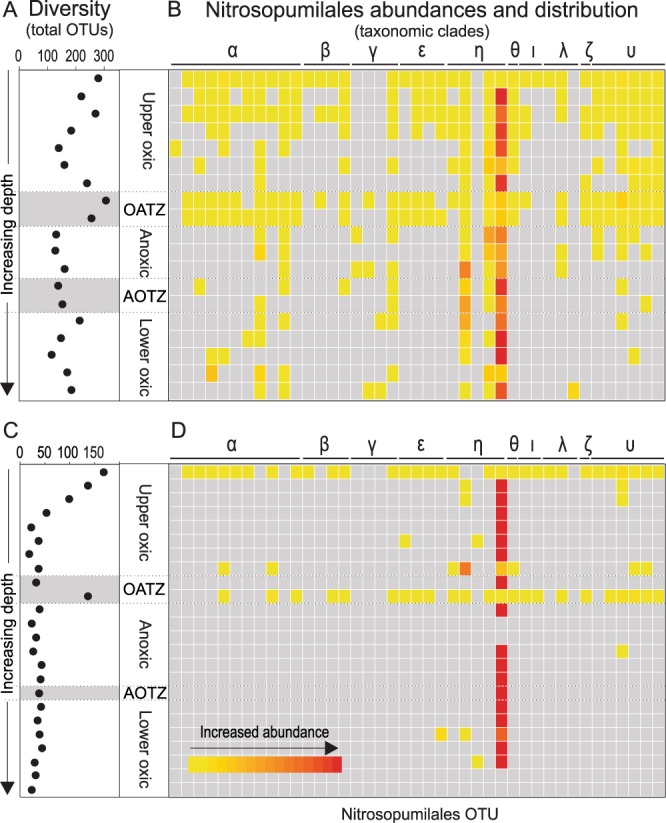
Microbial diversity and distribution of nitrifiers. Microbial richness as a function of depth measured by OTU abundance in core 3E (**A**) and 4A (**C**). The oxic/anoxic (OATZ) and the anoxic/oxic (AOTZ) transition zones are marked with grey. Heatmap of the relative abundances of different Nitrosopumilales species (OTUs) is represented by each individual column in core 3E (**B**) and 4A (**D**). Distinct phylogenetic clusters are marked with Greek letters.

When examining the OTU-level community structure of AOA as a function of sediment depth/age (Fig. 4B,D), we find that most OTUs located in the deeper sediments also occur in the surface layers, albeit at a much lower relative abundance. In addition, a marked increase in AOA diversity (richness) is found in the OATZs, with a taxonomic composition similar to the surface layer (Fig. 4B,D). Phylogenetic analysis revealed that members of the Nitrosopumilales Eta (η) cluster dominate within the AOA community in deeper horizons, whereas the surface layers and the OATZ contain a much higher diversity (Fig. 4C,D). Similar to the Nitrosopumilales, the overall microbial richness is highest in the surface sediment layer (280 at 3E and 169 at 4A) and decreases sharply downcore, but is interrupted by a marked richness increase in the OATZs at both sites, resembling that of the surface layer (Fig. 4A,C).

### Reaction rates

We used a reaction-transport model to simulate the profiles of oxygen and nitrate, and to model the reaction rates of nitrification, denitrification, and oxygen respiration simultaneously. The simulated profiles of oxygen and nitrate match well with those observed, and the predicted TOC is within the range of previous reports (0.02–0.3% (20); Fig. S1). Nitrification rates vary two to three orders of magnitude (4.1 × 10^−7^–2.5 × 10^−4^ in 3E and 1.3 × 10^−7^–3.4 × 10^−5^ mol m^−3^ yr^−1^ in 4A) and are higher in the upper and lower oxic zones than in the anoxic zone, while the opposite trend was predicted for denitrification rates (Fig. 1). Oxygen consumption rates show relatively high initial surface respiration followed by a slow but steady decrease with depth (Fig. 1). To evaluate whether or not denitrification is active in the oxygenated sediments, we performed a sensitivity test by varying the inhibition concentration of O_2_ (*h*_1_) over a wide range (0.01–50 µM) and found a best-fit *h*_1_ value of 10 µM (Fig. S2, Table S4), suggesting that the denitrifiers detected in the oxygenated parts of the sediments are actively performing denitrification.

**Figure 5:**
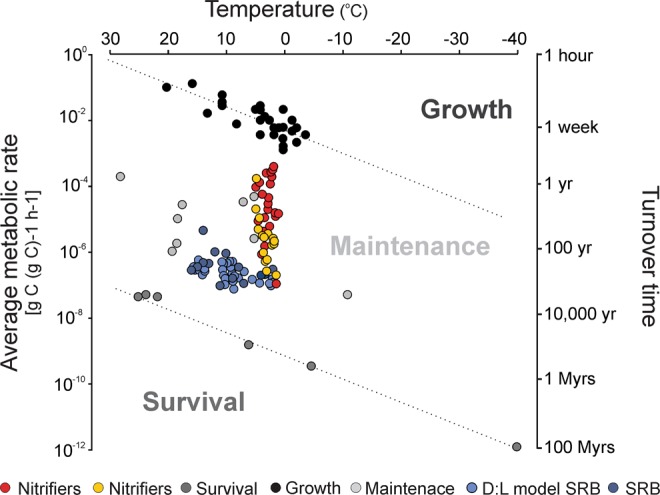
Mean metabolic rates and turnover times. Mean metabolic rates (left y-axis), converted from cell-specific rates, plotted against inferred temperature to identify the metabolic status. Turnover time of cellular carbon (right y-axis) as a function of temperature adopted from Price and Sowers (2004). Nitrifiers in core 4A (red) and 3E (yellow). Literature values from marine subsurface sulfate reducers are obtained from ref.^57^ (dark blue) and the D:L razemization model values from ref.^54^ (light blue). Metabolic rates needed for survival (grey circles), maintenance (light grey circles) and growth (black circles) are obtained from Price and Sowers (2004).

Calculated cell-specific oxygen consumption rates vary between 10^–5^–10^–2^ fmol e^−^ cell^−1^ d^−1^ similar to the cell-specific rates of nitrifiers and denitrifiers (Figs 1 and S3). We translated the cell specific rates of nitrifiers into carbon metabolic rates assuming one mole carbon is fixed at the expense of 10 moles of ammonium oxidized^30,31^ and 14 fg of weight for each cell^32^. These metabolic rates range between ~10^−7^–10^−4^ g C (g C cell)^−1^ hour^−1^ and characterize the nitrifiers as being in maintenance state with cell turnover times spanning approximately 3 orders of magnitude, from months to several hundred years (Fig. 5).

## Discussion

### Microbial ecology of the N cycling population

The near absence of detectable ammonium and the prevalence of nitrate in concentrations exceeding that of the overlying seawater (Fig. 1) are clear indicators of active nitrogen cycling within the sediments beneath North Pond. Moreover, a substantial fraction of the microbial population is inferred to be involved in nitrogen transformation processes, primarily nitrification. Our reaction rate estimates and the abundance of ammonia-oxidizers (Figs 1–3) point to nitrification as the overall most prominent nitrogen cycling process above and below the anoxic zone. Among the ammonia-oxidizing microbes, which mediate the first step of nitrification, Archaea (AOA) are highly dominant relative to their bacterial counterpart in all but one sample (Figs 2, 3). Specifically, the relative abundance of AOA belonging to the Nitrosopumilales is at least one order of magnitude higher than AOB (*Nitrosospira* and *Nitrosococcus*) (Fig. 3). This result might be attributed to a higher oxygen affinity of AOA having extremely low *K*_*m*_ values and a highly energy efficient CO_2_ fixation pathway^33^, which allows them to outcompete AOB in oxygen- and ammonium-limited regions where energy is low^11,34–36^. With respect to nitrification rates, however, some AOB are known to have higher metabolic rates than AOA^37^, implying that the relative contribution of AOB to overall nitrification might be higher than inferred from their abundance. Our community profiling suggests that the second step of nitrification, nitrite oxidation, is catalyzed by NOB affiliated with *Nitrospira* and *Nitrospina*. A tight coupling of ammonium oxidation and nitrite is suggested by dual isotopic analysis of pore-water nitrate at this site^12^. Hence, the absence of nitrite in the pore water could suggest that the catabolic activity of the NOB population is as high as the ammonia-oxidizers, irrespective of the significantly lower abundance of NOB, throughout both cores (Figs 2, 3). This inference would be in agreement with recent findings from the dark ocean indicating that NOB have a higher metabolic efficiency than the numerically dominant AOA^38^. However, we cannot exclude partial nitrite removal by denitrifiers, which would lead to overestimations of the NOB activity level.

Despite the difficulties of taxonomically identifying denitrifiers^39^, both the reaction-transport model results (Fig. 1) and the distribution of functional genes (Fig. 2) indicate that denitrifiers are present and active even in the oxic zones. While this result seemingly contradicts the traditional view that denitrification is limited to anoxic/hypoxic environments, it supports mounting evidence of denitrification in oxic marine sediments^40^. Whether this finding is best explained by the presence of anaerobic micro-niches or by oxygen tolerance is still unclear.

From surface to basement, the microbes involved in nitrogen transformation are taxonomically congruent with those found in surface sediments from other open ocean sites^13,41^, suggesting that a canonical assemblage of higher-rank microbial taxa regulate nitrogen cycling in the oligotrophic sedimentary realm. Beyond the community composition, however, the vertical distribution and abundances of these taxa shed light on processes and activity at depth. The occurrence of Nitrosopumilales 16S rRNA and AOA *amoA* genes at depths without detectable oxygen (Figs 2, 3) supports the inferred presence of this group in oxygen-deprived marine sediments^42–44^, and raises questions about their metabolic capabilities. To this end, our calculated nitrification rates in zones without detectable oxygen require only nanomolar levels of O_2_ (well below the analytical detection limit). Furthermore, with our estimated oxygen consumption rates, which are always higher than the nitrification rates (Fig. 1), the O_2_ flux should be sufficient to accommodate the nitrification process. Recent studies also show that pelagic AOA in oxygen minimum zones have extremely low *K*_*m*_ values of 333 nM O_2_^11^, and that oxygen concentrations less than 10 µM are sufficient to support the growth of specific AOA strains^45^. Hence, our data provide no evidence to suggest that AOA cells found in oxygen-deprived zones are dead, inactive, or involved in any other metabolism than aerobic ammonium oxidation.

### Cell-specific reaction rates, physiological status and turnover time

When considering the sedimentary community as a whole (excluding nitrifiers), and assuming it to be involved in aerobic heterotrophic metabolism, the cell-specific oxygen consumption rates, at North Pond fall within those estimated for communities at similar depths from oligotrophic and oxygenated sediments beneath the North and South Pacific gyres^17,46^ (Fig. S3). Our estimates help constrain the basal energy requirements for the community as a whole, indicating a lower energy limit around 10^−5^ fmol electrons cell^−1^ day^−1^, equivalent to a daily electron transfer on the order of 10^3^. However, such estimates assume equal activity and function for all members of the community and are thus unable to distinguish between different metabolic strategies, which could mask significant variation in the level of activity among microbial groups and individual cells. Although mounting evidence points toward a deep sedimentary community in which the majority of cells are metabolically active^47^, the specific minimum energy required to sustain a microbial cell is likely to depend on environmental conditions as well as differences in metabolic strategy e.g. autotrophy vs heterotrophy^48^.

Our data enable us to go beyond bulk respiration estimates, where all cells are treated as equal, and to resolve cell-specific rates of nitrogen transformation, specifically nitrification rates (Fig. 1). In contrast to the cell-specific oxygen consumption, which is relatively stable (roughly within one order of magnitude in each core), cell-specific nitrification rates are highly variable, spanning several orders of magnitude between sampled horizons. Despite very low metabolic activity levels, cell-specific nitrification rates at >10 mbsf depth are notably higher than the cell-specific oxygen respiration rates (Fig. 1C), and this pattern also holds true when cell-specific oxygen rates are corrected for cell variation in 16S rRNA gene copy numbers (Fig. S3). It is unclear if this relatively higher energy output is related to their autotrophic lifestyle and a potentially higher anabolic energy requirement. Nevertheless, the nitrification rates are many orders of magnitude lower than *in situ* rates estimated in surface environments, e.g. freshwater surface sediments [29–65 fmol cell^−1^ d^−1^ ^49^], as well as rates obtained from cultured representatives of AOB [24–550 fmol cell^−1^ d^−1^; Prosser^50^] and AOA [*Nitrosopumilus maritimus*, 4 fmol cell^−1^ d^−1^; Könneke, *et al*.^8^].

Our analysis also provides reliable estimates of total denitrification rates and predicts active denitrification throughout both cores (Figs 1–3). However, these inferred cell-specific rates (Fig. S3) have to be qualified, since denitrifiers are metabolically versatile and can switch electron acceptors, e.g. from oxidized nitrogen compounds to oxygen.

Transformation of cell-specific nitrification rates into carbon metabolic rate estimates categorize the nitrifiers as being in maintenance state (Fig. 5), in agreement with growing evidence that the majority of microorganisms present in deep marine sediments are viable but on physiological standby^46,51,52^. The recent proposal of a basal power requirement (BPR) that limits subseafloor microbial persistence is based on estimated aerobic heterotrophic respiration and sulfate reduction rates^46–48^ and our results suggest that the lower BPR limit (~10^−5^ fmol e^−^ cell^−1^ day^−1^) also holds true for autotrophic nitrifiers (Fig. 1). When translating these metabolic rates into turnover rates, generally they are within months to tens of years (Fig. 5), thereby lending support to the recent suggestion^53^ that turnover times could be significantly lower than previous assumed for the deep sedimentary population^54^. We note that the slowest turnover rates for nitrifiers are within the anoxic zones of our investigated cores.

### Local growth in redox transition zones

At depth greater than 20 mbsf the OATZs and the AOTZs have been isolated from fresh organic carbon input and surface community recruitment for several million years. Nonetheless, local peaks in archaeal and bacterial cell abundance at both the OATZs and the AOTZs (Fig. 2) indicate that these zones are able to sustain a greater biomass than adjacent horizons, in agreement with diagenetic model predictions of enhanced biological activity in sedimentary OATZs^55,56^. The motility of microbial cells in subseafloor sediments is considered to be limited by energy and space^48,57^. If so, and assuming steady surface input, then the observed abundance peaks can only be explained as a result of *in situ* growth. Local abundance peaks have previously been found in a sulfate-methane transition zone (SMTZ) in organic-rich sediments^4^. Taken together, these findings suggest that the increased energy available at redox transition zones where reduced and oxidized chemical species meet, such as the OATZ, AOTZ and SMTZ, allows starved cells to grow and divide *in situ*.

Recent advances in our basic understanding of microbial evolution and community assembly support the notion that microbes currently populating the deep sedimentary biome are direct descendants of a persistent subset of those cells once deposited on the seafloor^58,59^. Due to the highly energy limited conditions encountered at depth, a strong selective pressure favors the taxa able to survive the harsh conditions, leading to a general decrease in diversity with depth. Our results, however, add an important aspect to this general picture by finding that the *in situ* growth of nitrifiers at the OATZ is accompanied by an increase in diversity (observed OTU richness) and by a resurrected community structure resembling that of the surface layers (Fig. 4B,D). This pattern is most striking at site 4A, where most of the 31 AOA OTUs present in the surface become so rare that they are no longer detectable in the amplicon library, yet 75% of the OTUs reappear in the OATZ (Fig. 4B). This finding strongly suggests that the increased diversity is a result of *in situ* growth rather than of fast evolution (diversification) of abundant members, congruent with the recent finding that genomic evolution in subseafloor sediments is negligible^58^.

An increase in nitrifier abundance at the AOTZs (e.g. Fig. 2) indicates that this boundary also has a higher energy flux, which the ammonia oxidizers can tap into. However, the absence of a concomitant community resurrection at the AOTZs (Fig. 4B,D) suggests that only the most persistent taxa survive the protracted transition through the anoxic zone and are able to benefit from the increased energy availability. This ability to rebound at the OATZ but not at the AOTZ seems to hold true for the community at large and not only for nitrifiers as apparent from the overall microbial diversity (Fig. 4A,B) and the community clustering pattern (Fig. S4).

### Nitrogen fluxes

Oligotrophic conditions at North Pond cause an imbalance in the nitrification-denitrification rates, which we observe as nitrate accumulation in the sediment pore water. This in turn leads to an efflux of nitrate both into the overlying seawater and into the underlying oceanic basement (Table S1). The upward nitrate efflux (0.03–0.05 mmol m^−2^ y^−1^) is in agreement with the general understanding of deep-sea sediments acting as a source of dissolved nitrogen for the overlying water masses^60,61^. We note that although our upward nitrate efflux estimates for the topmost active layer are probably biased due to the relatively low sampling effort in the surface sediments, they are comparable to those reported from the oligotrophic South Pacific Gyre^18^. Moreover, we estimate that 19–24% of total sedimentary nitrate efflux diffuses into the underlying oceanic crust (Table S1), which matches exactly the range estimated from the oligotrophic sediments at the Clarion-Clipperton fraction zone^20^. This downward supply of sedimentary nitrate could be important for sustaining microbial life in the crustal habitat as indicated by incubation experiments^62^, and by the vast nitrogen cycling potential of microbes in both the crustal fluids and hard rocks^63–65^.

**Figure 6:**
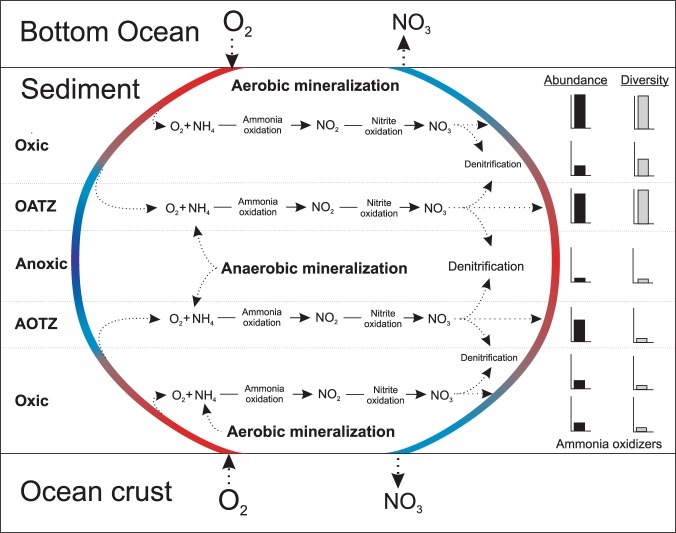
Schematic summary of nitrogen transformation and nitrifying community changes in oligotrophic sediments with a hydrologically active basement. Oxygenated seawater in the bottom ocean and in the crustal aquifer cause an influx of oxygen into the sediment column from above and below. Rapid mineralization of organic carbon in surface sediments releases ammonia, which is instantly consumed by nitrifiers, supporting their high initial abundance and diversity. Below the high-activity surface layers, nitrifier abundance and diversity decline due to restricted organic carbon mineralization and thereby limited ammonia availability. In the oxic-anoxic transition zone (OATZ), ammonia is made available again through diffusion from the deeper anoxic layers that support anaerobic mineralization and release of ammonia, causing an increase in nitrifier cell abundance and diversity in the OATZ. In the anoxic zone nitrification is inhibited and both the diversity and abundance of nitrifiers decreases. When entering the anoxic-oxic transition zone (AOTZ), nitrifiers are no longer oxygen limited, and we detect an increase in abundance but not in diversity. As the nitrifiers leave the AOTZ, their abundance declines and their diversity remains low. Although denitrification occurs throughout the sediment column, limited organic carbon availability is likely responsible for maintaining the rates of denitrification lower than the production of nitrate, which results in an efflux of nitrate into the overlying bottom ocean and into the underlying crust.

To summarize, a conceptual model of the nitrogen cycling processes and the associated community dynamics in the oligotrophic sediments of North Pond is provided in Fig. 6. The imbalance of metabolic activities of nitrifiers and denitrifiers maintain high nitrate concentrations throughout the sediment column, and is likely caused by limited carbon availability for denitrifiers. The result is a nitrate efflux into the overlying seawater and the underlying basaltic aquifer, both habitats where nitrate may act as a limiting nutrient^62,66,67^. Our calculated cell-specific rates of nitrification and denitrification in the oxygenated sediments are comparable to previously obtained aerobic bulk respiration rates from similar environments in the North and South pacific gyres. However, we note that our nitrification rates are generally higher than the estimated aerobic respiration rates, potentially as a consequence of the nitrifiers’ autotrophic lifestyle. Overall, nitrifiers are in a maintenance state, with turnover times varying between months to hundreds of years. We highlight the OATZ as a microbial hotspot of subsurface nitrogen transformation, where reduced nitrogen (ammonia) diffuses up from deeper anoxic layers and provides additional substrate, which otherwise is limited. This redox transition zone hosts a higher abundance of cells and a greater microbial diversity, particularly of nitrifiers, relative to adjacent horizons. We argue that these isolated peaks serve as indicators of local microbial growth, even though these cells have been sequestered from fresh organic matter input for millions of years. The microbial community composition in the OATZ suggests that the original surface nitrifying community has been resurrected at depth after a protracted period of dormancy. Despite a suggestive increase in AOA cell abundance at the AOTZ, the absence of a comparable diversity peak implies that the majority of the nitrifying community cannot rebound after passing through the anoxic zone.

## Materials and Methods

### Sample location and collection

The two cores used in this study were obtained during the Integrated Ocean Drilling Program (IODP) Expedition 336 to North Pond, an isolated small sediment pond (8 km × 14 km) above basaltic ocean crust located on the western flank of the Mid-Atlantic Ridge at 22°49′N; 46°05′E. The water depths of the two coring sites were 4425 (U1383E) and 4475 (U1384A) meters below sea level. Temperature of the bottom seawater is assumed to be 1.5 °C^25^. The cores were cut into 1.5 meter-long sections on deck and immediately sampled for microbiological and sediment pore-water analyses, as described elsewhere^23^. For this study, sediment from the interior part of intact whole cores was sub-sampled using autoclaved cut-end 10 ml plastic syringes and stored in sterile Whirlpak® bags at −80 °C until further analysis. We note that a few horizons at site 3E showed signs of weak to moderate coring disturbance^19^.

### Flux calculations

Based on the pore-water profiles of oxygen reported in Orcutt, *et al*.^25^ and nitrate profiles reported in^19,56^, the diffusive fluxes of nitrate between sediments, the overlying seawater and the underlying basement, as well as fluxes of oxygen and nitrate in and out of the oxic-anoxic and anoxic-oxic transition zones were calculated using Fick’s first law of diffusion (details can be found in SI). Due to relatively low spatial resolution of data points in the surface sediments where the most radical curvature of geochemical profiles occur, the values reported should be regarded as minimum estimates.

### Reaction-transport modeling

Geochemical interactions for the entire sedimentary sequence at both sites were simulated using a one-dimensional reaction-transport model^20,68^, which discretizes the advection-diffusion-reaction equation. The model used in this study considers three primary reactions: aerobic respiration (*R*_1_), heterotrophic denitrification (*R*_2_), Manganese reduction (*R*_3_); and two secondary reactions: nitrification (*R*_4_) and Mn^2+^ oxidation (*R*_5_), between six chemical species (organic matter, O_2_, NO_3_^−^, NH_4_^+^, Mn^2+^, and MnO_2_). The model simulations assume that the geochemical profiles, including all implicit reactive intermediates, are near steady state. For details see SI.

### DNA extraction

A total of 43 sediment horizons were selected for DNA extraction (~3 meter intervals) from sites 3E (19 horizons) and 4A (25 horizons). Genomic DNA was extracted using PowerLyse^®^ DNA Isolation Kit (MOBIO Laboratories, Inc.) following the manufacturer’s instruction with two modifications. First, the special bead coating G2 DNA/RNA enhancer (Ampliqon A/S, Odense, Denmark) was used as described in Bælum *et al*.^69^. Second, 200 μg of sterile filtered polyadenylic acid^70^ (Sigma) was added to each lysis mixture prior to bead beating (Hugenholtz *et al*. 1998). Bead beating was performed using the MP-Biomedical FastPrep**®**-24 for 45 seconds (speed setting 6). To track potential contaminants introduced during the drilling and experimental processes, DNA from the drill mud, the plastic bag carrying the fluorescent microspheres, and the kit reagents were also extracted as describe elsewhere^71^. DNA extracts from each sample was finally eluted into 100 μl PCR-grade double-distilled water (ddH_2_O), and preserved at −20 °C until further analysis.

### PCR screening of functional genes

The functional genes encoding the key enzymes of the following nitrogen transformation processes were screened using conventional PCR in all 43 horizons: archaeal and bacterial ammonium oxidation (AOA *amoA* and AOB *amoA*, respectively*)*, nitrite oxidation (*nxrB)*, nitrate reduction *(narG* and *napA)*, nitrite reduction (*nirS* and *nirK)*, nitrous oxide reduction (*nosZ*), anaerobic ammonium oxidation (*hzsA* and *hzo)*, dissimilative nitrate reduction to ammonium (*nrfA)*, and nitrogen fixation (*nifH*). In addition we screened for the presence of sulfate reducers, by targeting the *dsrB* marker gene. A complete list of primers and specific PCR conditions can be found in Table S5. Each reaction (25 µl total volume) contained the following: 1× HotStar Taq® Master Mix (Qiagen, Hilden, Germany), 1.2 µM of each primer and 1 µl template DNA. PCR amplification for each gene was performed for 40 cycles, and products evaluated by visual inspection on 1% agarose gels.

### Enumeration of gene abundance by quantitative PCR

Based on initial PCR screening, the six functional genes successfully amplified in at least one of the 43 sediment horizons (i.e. AOA *amoA*, AOB *amoA*, NOB *nxrB*, nitrate reducer *narG*, as well as denitrifier *nirK* and *nirS*) were subsequently enumerated by quantitative PCR (qPCR). In addition, archaeal and bacterial 16S rRNA gene abundances were quantified as described elsewhere^43^. All standards were quantified using BIO-analyzer (DNA 1000 chips, Agilent Technologies) and 10-fold serial diluted to 10–10^6^ copies µl^−1^. Details can be found in SI, including a complete list of primers and thermal conditions Table S5.

### Cell specific reaction rates and metabolic activity

Mean cell-specific rates of nitrifiers and denitrifiers were calculated by dividing the total volumetric reaction rates predicted from the reaction-transport model by the total abundance of the respective functional groups measured by qPCR (details in SI). Due to the possible presence of multiple copies of a given functional gene in one genome, the calculated cell-specific rates should be considered minimum values. The cell-specific rates were normalized to femtomoles of electrons (e^−^) transferred per cell per day, under a number of assumptions, which can be found in the SI. For comparison with other relevant studies, cell specific reaction rates were recalculated into carbon metabolic rates as described in the SI.

### 16S rRNA gene libraries and sequencing

DNA extracted from site 3E and 4A (43 horizons in total) was amplified in duplicate reactions using a previously described two-step amplification strategy, minimizing amplification bias^43,72^. 16S rRNA genes were PCR amplified using primers Uni519F (5′-CAGCMGCCGCGGTAA-3′) and 1392 R (5′-ACGGGCGGTGWGTRC-3′) for samples from 4A, and Uni519F and 806 R (5′-GACTACHVGGGTATCTAATCC-3′) for 3E, to create the initial amplicon libraries. More details can be found in the SI section.

### Taxonomic evaluation and functional classification

After quality control (details in SI) the filtered reads were clustered into Operational Taxonomic Units (OTUs) at 97% nucleotide similarity cutoff using UPARSE^73^. Taxonomy of the representative sequences of each OTU were assigned using the software packages CREST against the SilvaMod reference database^74^ using a common ancestor algorithm. OTUs with taxonomic affiliation relevant to nitrogen transformation were manually assigned into functional groups as follows: the order Nitrosopumilales was assigned into ammonium oxidizing archaea (AOA), the genus *Nitrosospira*, *Nitrosomonas* and *Nitrosococcus* were considered ammonium oxidizing bacteria, AOB, the genus *Nitrospira* and *Nitrospina* were treated as nitrite oxidizing bacteria (NOB), and denitrifying bacteria i encompass the taxa of *Pseudomonas*, *Arcobacter*, *Aeromonas*, and *Woeseiaceae* (Tables S6, S7). OTUs of Nitrosopumilales were extracted from the decontaminated OTU table, and the relative abundance of each OTU in the total AOA communities in each core was normalized by total-sum-scaling before displayed in the heatmap (Fig. 4) prepared using the R package ggplot2^75^. For diversity measures (richness) each sample was randomly subsampled to 1,000 reads in USEARCH v10^76^, prior to any comparison between samples.

## Supplementary information

supplementary information, figures and tables

## Data Availability

Raw read files generated in this study have been deposited in the NCBI Sequence Read Archive under accession number PRJNA489438. Geochemical data related to the two investigated cores can be found at IODP Expedition 336 webpages (http://publications.iodp.org/proceedings/336/336toc.htm).
