# Supplementary material for: Nitrifier abundance and diversity peak at deep redox transition zones
Source: Sci Rep. 2019 Jun 14;9:8633. doi: 10.1038/s41598-019-44585-6 (PMC6572806; doi:10.1038/s41598-019-44585-6)
Supplement: Supplementary file 1 — supplementary information, figures and tables [file 41598_2019_44585_MOESM1_ESM.pdf]

Title

Nitrifier abundance and diversity peak at deep redox transition zones

Authors

Rui Zhao[1,2,†,*], Bjarte Hannisdal[2], Josè M. Mogollon[3] & Steffen L. Jørgensen[2,*]

Affiliations

[1] Department of Biology, University of Bergen, Bergen, Norway
[2] K.G. Jebsen Centre for Deep Sea Research, Department of Earth Science, University of Bergen, Bergen, Norway
[3] Institute of Environmental Sciences (CML), Leiden University, Leiden, Netherlands
[†] Present address: School of Marine Science and Policy, University of Delaware, Lewes, DE, 19958, USA

[*] **Correspondent authors:** steffen.jorgensen@uib.no or ruizhao@udel.edu

Supplementary Materials

Flux calculations

Fluxes were calculated using Fick's first law of diffusion: $J = \varphi \, x \, D_s \, x \, \delta[O_2]/\delta z$

Where, J is the flux; φ is the measured sediment porosity; D_s is sedimentary diffusion coefficient for solutes (m^2 yr^{-1}) calculated using the R package *marelac* [1]; z is the sediment depth below the seafloor (m); and $\delta[O_2]/\delta z$ equals the change in solute concentration gradient (mmol m^{-3}), which were calculated from the nearby three data points.

Quantitative PCR (qPCR)

All qPCR assays were performed on a StepOnePlus real-time PCR platform (Life Technologies, Carlsbad, CA, USA) using the QuantiTect SybrGreen PCR kit (QIAGEN). Each reaction (20 µl) contained 1× QuantiTech SybrGreen PCR master mixture, 0.5 µM forward and reverse primer and 1 µl DNA template. For all qPCR assays, product specificity was verified via dissociation curves, and multiple blanks were included in each run (none of which amplified). Detection limit was 10 gene copies per reaction. Quantification efficiency was estimated to 85-98% and all r values were above 99%. Samples and standards were analyzed in triplicate. Results were normalized to copy numbers per gram wet sediment (copies g^{-1} ws) for all genes analyzed.

Standard curves for archaeal 16S rRNA and AOA *amoA* genes were obtained using the AOA fosmid 54d9 [2]. For bacterial 16S rRNA genes, the standard used was genomic DNA obtained from *Escherichia coli*. For the reminding genes, PCR amplicons were obtained using environmental DNA as template using primers and thermal conditions identical to those used in

the PCR screening. Environmental DNA used for standard preparation was obtained from the following locations: AOB *amoA*, NOB *nxrB*, and denitrifier *narG* genes from North Pond sediment (site 4A, section 1H-1), *nirS* and *nirK* from Arctic permafrost soil (Courtesy of Dr. Antje Gittle). Resulting amplicons were cloned using the StrataClone PCR Cloning Kit (Agilent Technologies, USA) according to the manufacturer's instructions. Positive clones were amplified using the vector primers M13F/M13R, to generate linear qPCR standards. All standard DNA concentrations were measured using Bio-analyzer (DNA 1000 chips, Agilent Technologies) and serial diluted to 10-10^6 copies μl^{-1} for subsequent quantification.

Preparation of Amplicon libraries

The amplification of 16S rRNA genes was performed in duplicate reactions using the primers Uni519F (5'-CAGCMGCCGCGGTAA-3') and 1392R (5'-ACGGGCGGTGWGTRC-3') for samples from 4A, and Uni519F and 806R (5'-GACTACHVGGGTATCTAATCC-3') for 3E. Each reaction (25 µl) contained the following: 1× HotStar Taq® Master Mix (Qiagen, Hilden, Germany), 1.2 µM of each primer and 2 µl template DNA. PCR thermal condition was as follows: 95 °C for 5 min in the initial denaturation and polymerase activation, followed by 25-33 cycles of 95 °C for 30 s, 58 °C for 45 s, 72 °C for 1 min, and a final elongation of 72 °C for 10 min. The PCR cycle number at this stage was optimized individually for each sample in order to minimize PCR bias (Table S4). The duplicate PCR products were then pooled and purified using GenElute PCR Clean-up kit (Sigma-Aldridge). In order to add the adaptor sequences, a second-round duplicate PCR was performed (7 cycles) under the same thermal condition with the same primers as the first-round PCR but elongated with adapters and the forward primer containing a 12-bp error-correcting barcode unique to each sample [3]. Resulting amplicons were purified again, using AMPure XP (Beckman Coulter) following the manufacturer's protocol (bead-to-sample ratio 7:10) and the concentration and quality were controlled by 1% agarose gel inspection, Quantus Fluorometer (Promega) and Bio-analyzer (DNA 1000 chips, Agilent Technologies). All purified amplicons were pooled in a 1:1 ratio based on DNA concentration and sequenced using multiplex GS FLX plus pyrosequencing (flow pattern A) for the samples in 4A, and using Ion Torrent Personal Genome Machine (PGM) (Life Technology, USA) for the samples in 3E.

Filtering and noise removal from amplicon sequencing data

The quality filtering and clustering of reads were performed using the USEARCH and UPARSE packages [4,5]. Firstly, reads in each sample were quality filtered and trimmed to 220 bp using the '-fastq_filter' command using options '-fastq_trunclen 220' and '-fastq_maxee 1'. Chimeric

sequences were detected and removed with the '-uchime_ref' command using the Gold database as reference (available from 'http://drive5.com/uchime/gold'). A total of 780,489 sequences from site 3E and 179,693 sequences from 4A were available for further down-stream analysis after initial filtering.

De novo OTU clustering was performed at a cutoff of 97% nucleotide sequence similarity using the '-cluster_otus' command in UPARSE. Potential contaminants (i.e. OTUs detected in the drill mud, fluorescent microsphere bag, and blank kit extractions) in the datasets were removed using the strategy described in [6].Taxonomic classification of OTUs was performed using the program CREST with the SilvaMod reference database [7], applying the Lowest Common Ancestor algorithm. All samples were down sampled to 1,000 reads prior to diversity (richness) comparison between samples (Fig. 4a, c).

Clustering analysis

The similarity of samples of different depths in each core were calculated in the R package *Vegan* [8], using the Bray-Curtis distance, based on the relative abundances of OTUs, and visualized using the R package *dendextend* [9].

Reconstruction of phylogenetic trees

To verify the taxonomy of the OTUs of putative AOB and NOB, OTUs from each functional group were aligned in MAFFT [10], along with the 16S rRNA gene sequences of cultured species and their closest relatives from the NCBI database. Maximum-likelihood phylogenetic trees were reconstructed using RAxML [11], by applying the Generalized Time-Reversible model and the gamma approximation as the rate heterogeneity model (GTRGAMMA). Support values were determined using 100 non-parametric bootstrap replicates (Fig. S5a, b).

Reaction-Transport modeling

The model simulations assume that the geochemical profiles, including all implicit reactive intermediates, are near steady state. It includes the effects of compaction via an exponentially decreasing porosity equation, and biologically induced mixing via diffusive (bioturbation) and nonlocal (bioirrigation) transport terms, however the latter two can be disregarded in this study. Organic matter is assumed to consist of a continuum of reactive components (the reactive continuum model [12]). Aerobic respiration (R_1) was considered as the most favorable pathway of organic matter consumption. The inhibition concentration of O_2 for heterotrophic denitrification (h_1) was set to vary over a wide range (from 10 nM to 50 μM). Organic matter degradation

coupled to manganese oxide reduction (R_3) was inhibited by denitrification. The two secondary reactions, namely nitrification (R_4) denotes the complete oxidation of NH_4^+ to NO_3^-, and Mn oxidation (R_5), was represented through bimolecular kinetics. The five reactions between the six species are listed in the Supplementary Table S7. The C/N stoichiometry of the degraded organic matter was assumed to be similar to previous reports from the shallow sediment (~10; Ziebis, et al. [13]). The boundary conditions at the sediment-water interface (SWI) and sediment-basement interface (SBI) were taken from previous reports [13,14] (Table S2). The model parameters (Table S3) were evaluated and optimized by comparing the model results to the measured profiles of organic matter, oxygen, and nitrate.

Cell-specific reaction rates calculation

Cells-specific reaction rates were calculated for aerobic heterotrophs, nitrifiers, and denitrifiers, by diving the estimated bulk volumetric reaction rates by the corresponding abundances of the relevant functional genes quantified by qPCR.

This calculation was made under the following two critical assumptions: 1) the functional gene abundance represents the quantity of intact cells; and 2) all cells are equally active and differences in kinetics due to variation in substrate availability at different depths are negligible. While it is difficult to validate these assumptions, we note that the oxic nature of the environment makes it unlikely that extracellular DNA will persist over longer periods of time. We also note that the narrow range of substrate concentration and extremely low diversity of microbial communities, e.g. one Nitrosopumilales OTU detected in most of the deeper horizons at 4A, legitimate comparable metabolic kinetics between nitrifying cells, as also justified by Jørgensen and Marshall [15].

The abundance of nitrifiers, for each depth, was taken as the sum of AOA and AOB *amoA* gene copies. The number of *narG* gene copies represented denitrifier abundances. While this gene is also involved in the first step in the DNRA process, the lack of any apparent DNRA utilizes in the community profile and the unsuccessful detection of the diagnostic gene *nrfA,* catalyzing the second step lead us to argue that our assumption is reasonable. Aerobic heterotrophs were represented by the total cell abundances (archaeal + bacterial 16S rRNA gene copies) and subtracting the putative autotrophic nitrifiers (AOA, AOB, and NOB). Although this might still overestimate the abundance of heterotrophic cells, the bias inflicted by this assumption is likely to be less than one order of magnitude. It is also worth noting that the bulk oxygen respiration rates reported here only account for the part consumed by aerobic organic matter degradation i.e. it does not include the oxygen consumption by nitrification. We also include a calculation of the

cell specific oxygen consumption, which have been corrected for potential differences in gene copy numbers (assuming one copy for Archaea and 3 for Bacteria) and is displayed in Fig. S3.

All cell-specific rates were normalized to femtomoles of electrons (e^-) transferred per cell per day, assuming that six electrons are accepted by oxidizing a molecule of NH_4^+ to NO_2^-, five electrons are donated to denitrifiers during reduction of NO_3^- to N_2. In addition, four electrons are accepted by reducing a molecule of O_2, following ref. [16].

Cell-specific rates of nitrifiers were translated into cell-specific carbon metabolic rates (in unit of g C $(g\ C)^{-1}\ h^{-1}$), assuming that one mole carbon is fixed at the expense of 10 mole of ammonium oxidized [17,18], and that each cell on average weights 14 fg.

Supporting Figures

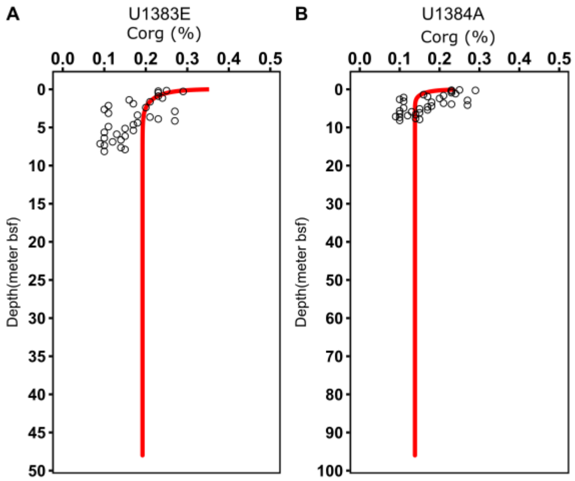

Fig. S1. Organic carbon content. Circles represent measured values of organic carbon in gravity core GeoB13507 from North Pond [13], while lines denote the simulated values in the sediments from site 3E (A) and 4A (B).

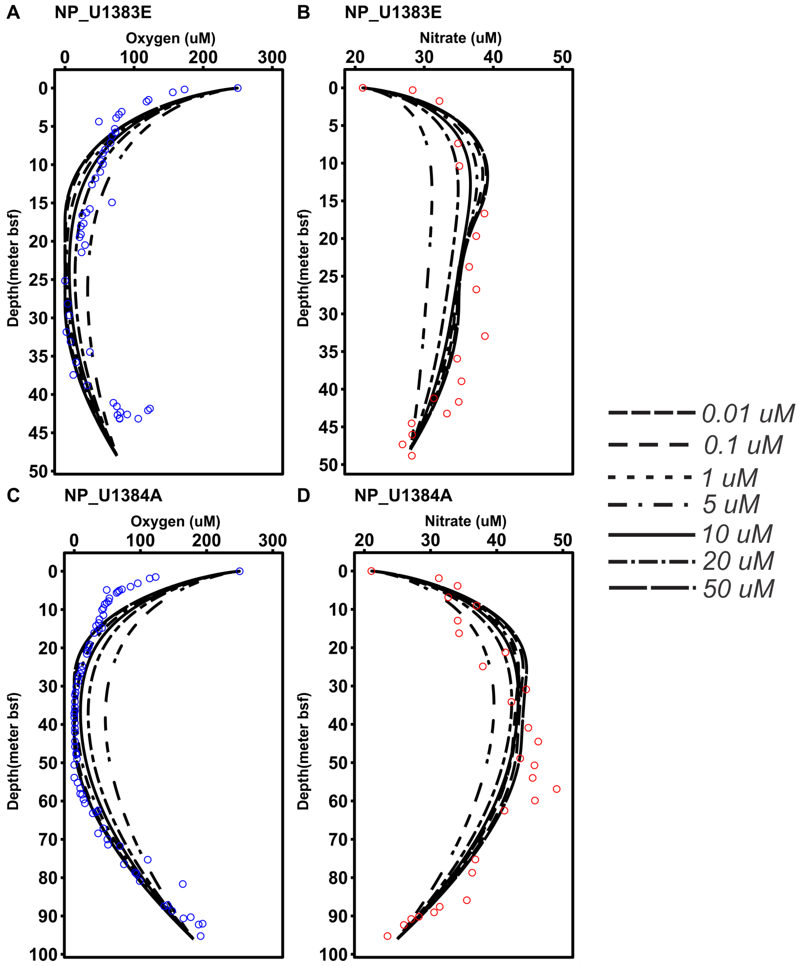

Fig. S2. Simulated geochemical profiles. Showing oxygen (A, C) and nitrate (B, D) when different inhibition concentrations of O_2 were used. Circles are measured values and lines represent the simulated values with different inhibition concentrations of O_2 (0.01 – 50 μM) while all other parameters were kept constant.

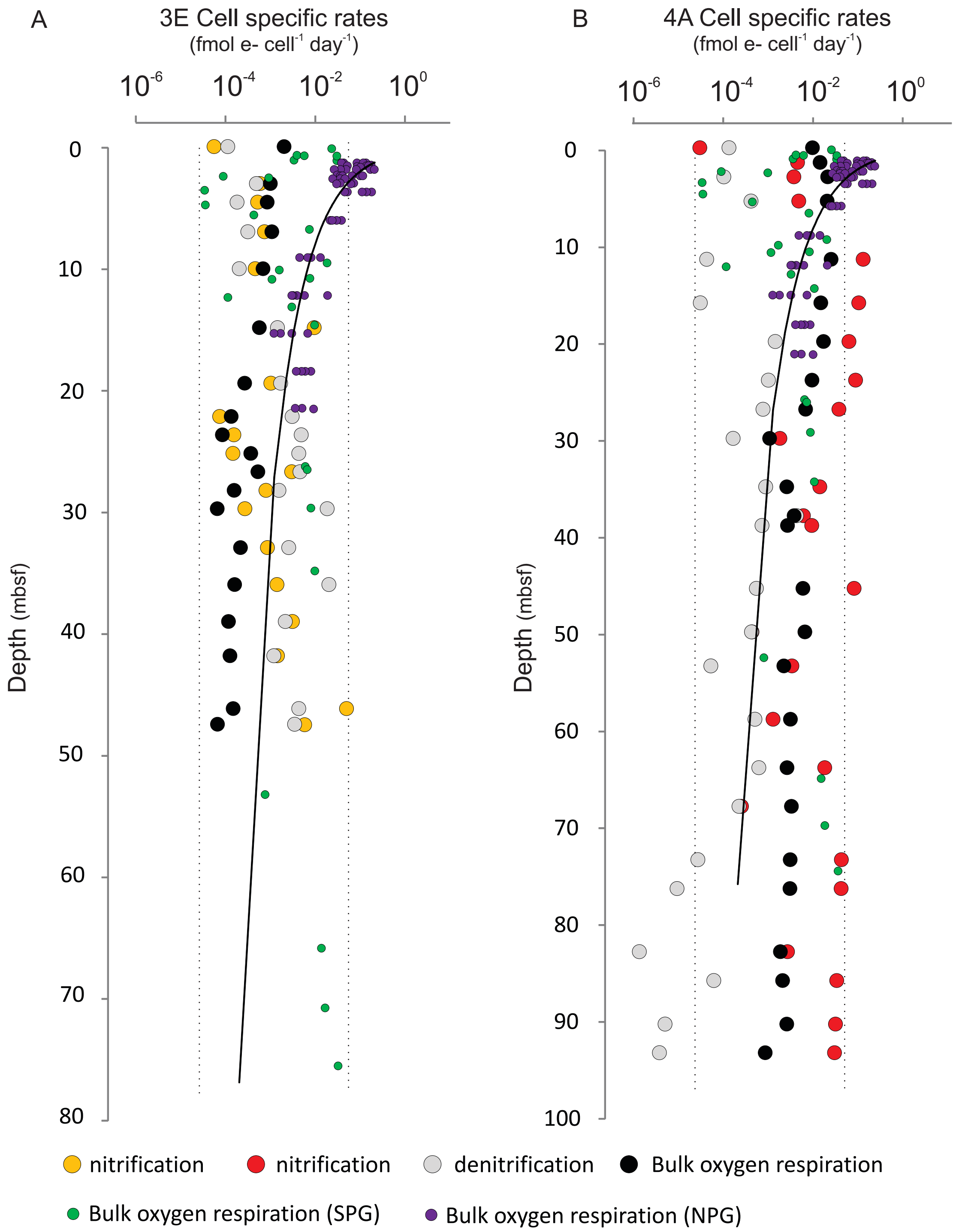

Fig. S3. Cell specific reaction rates. Estimated cell specific reaction rates at site 3E (A) and 4A (B) for nitrification (yellow and red at site 3E and 4A, respectively), bulk oxygen consumption (black) and denitrification (grey). In addition bulk oxygen respiration estimates from the North Pacific Gyre (NPG) (Røy et al., 2012) is added (purple) along with a power-law trend-line for this data (solid line). Further, bulk oxygen respiration estimates from the South Pacific Gyre (SPG) (D´Hondt et al., 2015) are implemented (green circles) and the upper and lower boundary for this dataset is highlighted (vertical dashed lines). Note that cell specific oxygen respiration values are corrected for multiple 16S rRNA copies per genome as opposed to that displayed in Fig. 1.

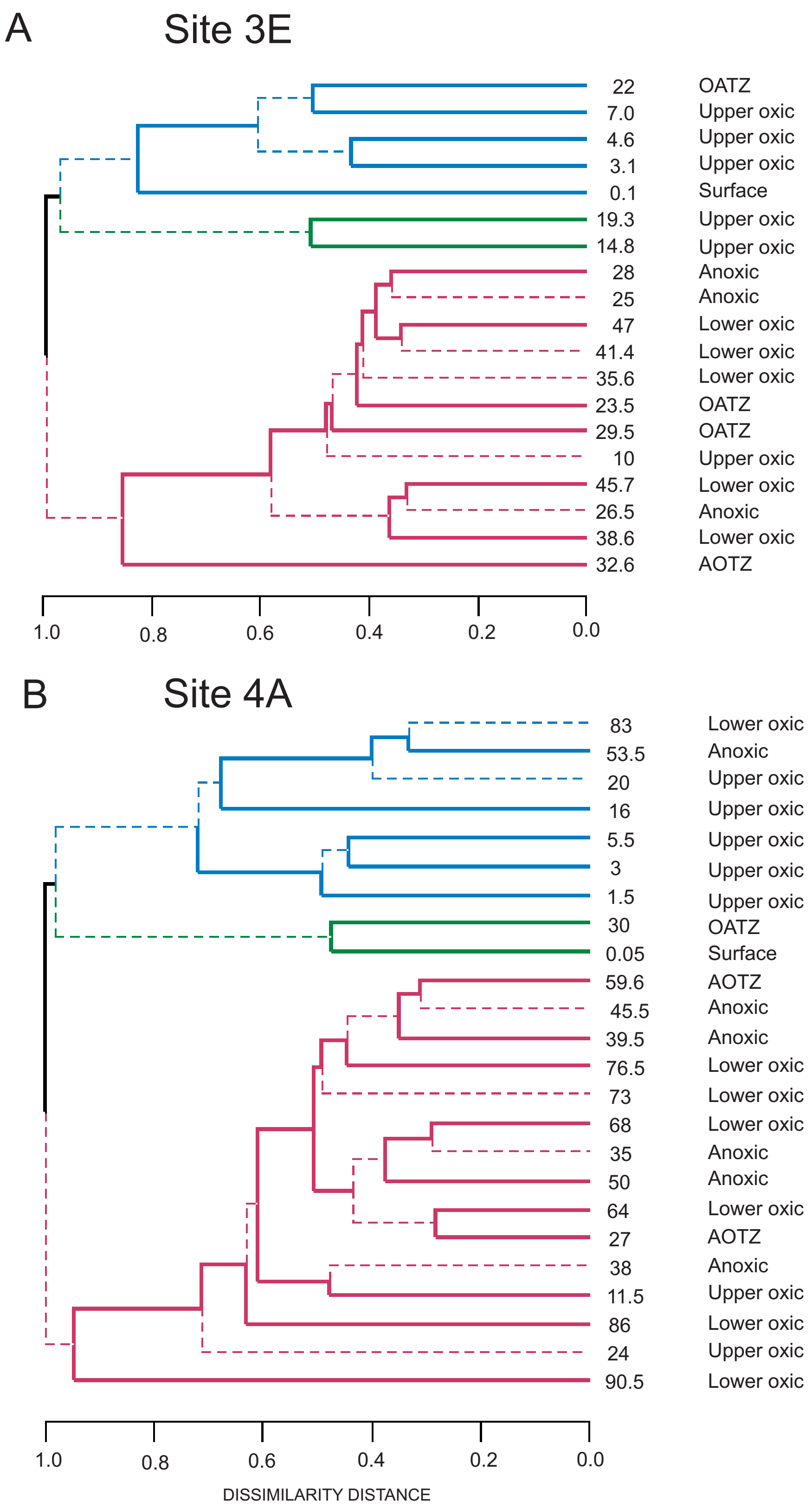

Fig. S4. Cluster analysis of total community structure. Bray-Curtis dissimilarity distances based on the relative abundance of each OTU at site 3E (A) and 4A (B). Different colors identify significant clustering (p = 0.05). Number at end of branches is depth in meters below seafloor. Dashed lines represent distinct branches. Oxygen regimes are indicated after each branch and divided into an upper and lower oxic zone, the anoxic zone, the oxic-anoxic transition zone (OATZ) and the anoxic-oxic transition zone (AOTZ).

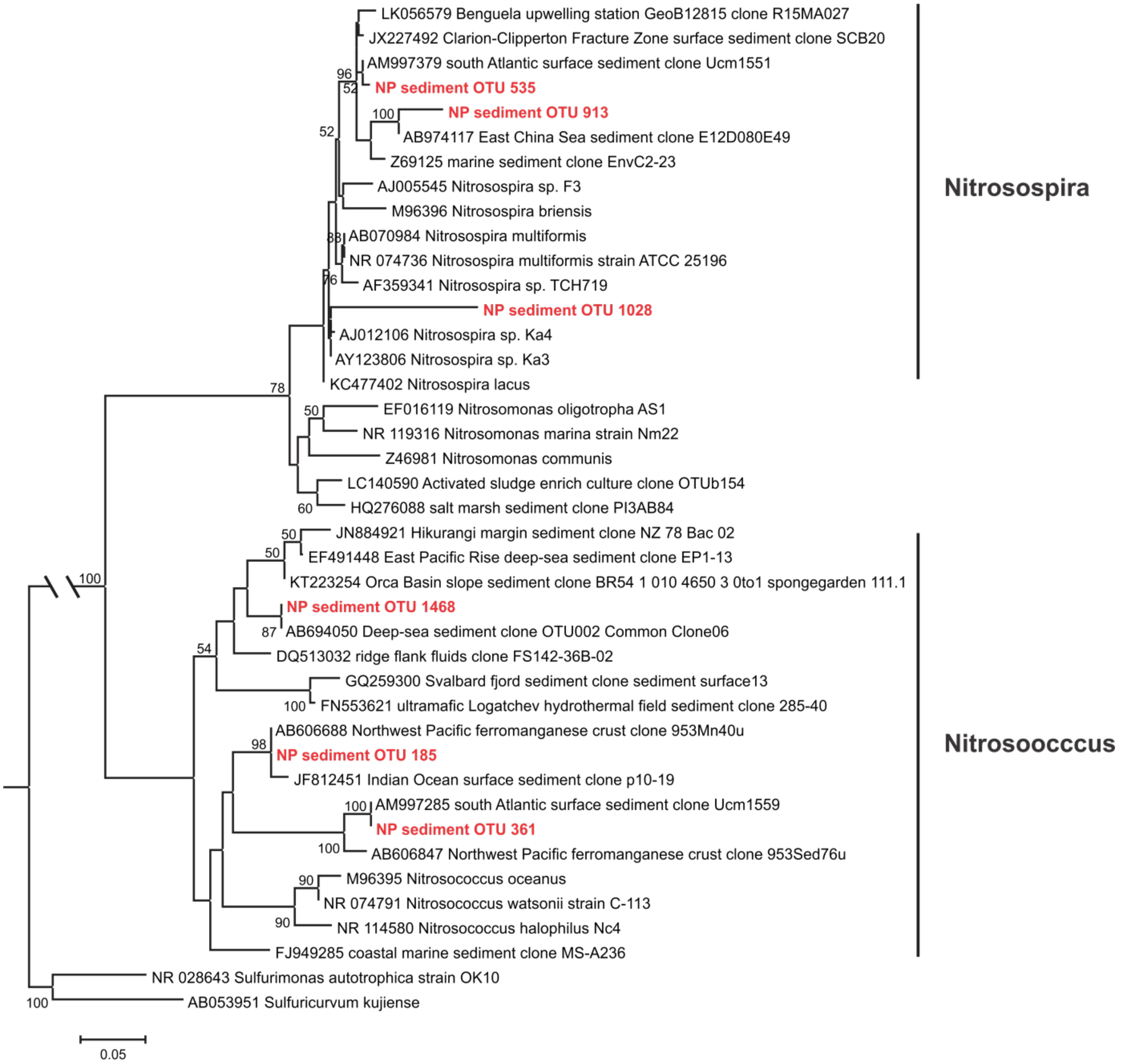

Figure S5a. **Phylogenetic tree of Ammonia-Oxidizing Bacteria**. Representative OTUs, based on partial 16S rRNA gene sequences, recovered from North Pond are shown in bold red and isolated strains shown in bold. The tree was reconstructed using the maximum-likelihood algorithm implemented in RAxML, by applying the Generalized Time-Reversible model and the gamma approximation as the rate heterogeneity model (GTRGAMMA).

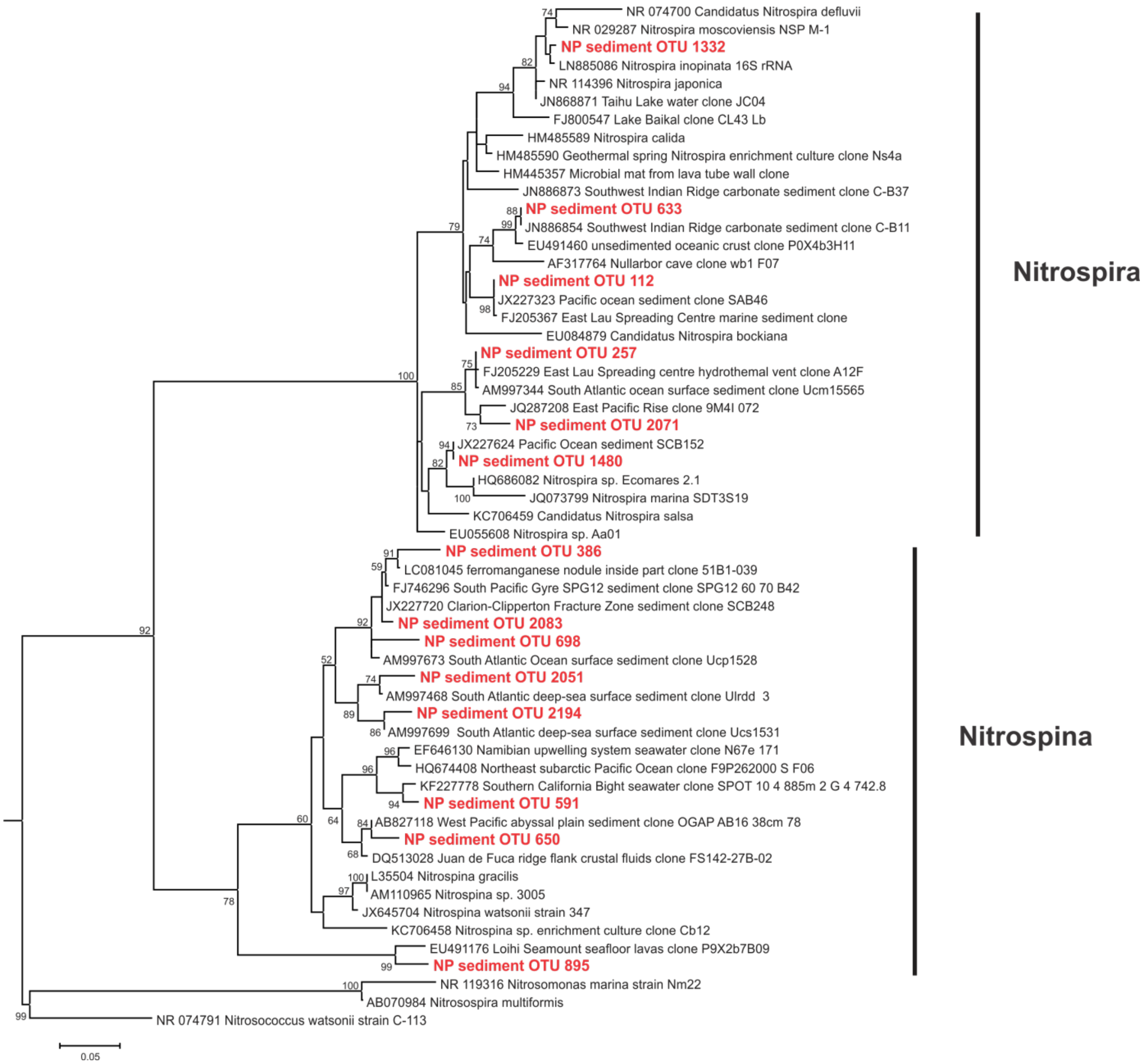

Figure S5b. **Phylogenetic tree of Nitrite-Oxidizing Bacteria**. Representative OTUs, Based on partial 16S rRNA gene sequences, recovered from North Pond are shown in bold red and isolated strains shown in bold. The tree was reconstructed using the maximum-likelihood algorithm implemented in RAxML, by applying the Generalized Time-Reversible model and the gamma approximation as the rate heterogeneity model (GTRGAMMA).

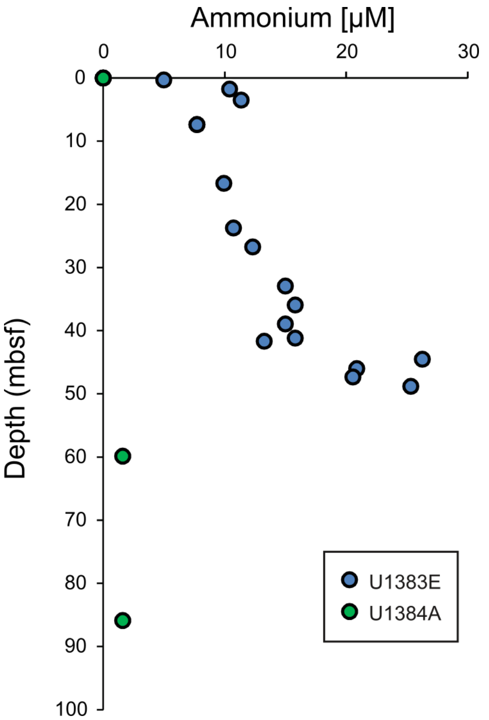

Fig. S6. Porewater ammonium profiles. Data from the two drill sites are from Ref. (19).

Supporting Tables

Table S1. Fluxes of oxygen and nitrate

Site	Sediment thickness (meter)	OATZ[a] depth	AOTZ[b] depth	Oxygen flux (mmol m^{-2} yr^{-1})				Nitrate flux (mmol m^{-2} yr^{-1})	
				Influx[c] (seawater)	Influx[c] (basement)	consumed (OATZ)	consumed (AOTZ)	efflux (seawater)	efflux (basement)
3E	43.3	21-25	28-33	1.0	0.09	n.a.[d]	0.04	0.047	0.011
4A	94.7	26-30	54-58	0.2	0.07	0.008	0.02	0.031	0.010

[a]: OATZ: Oxic-Anoxic Transition Zone.
[b]: AOTZ: Anoxic -Oxic Transition Zone.
[c]: data from Orcutt et al. (2013).
[d]: not available (low spatial resolution of oxygen measurement).

Table S2. Chemical reactions and associated rate expressions

Name	ID	Reaction	Rate expression	Rate Unit
Organic matter degradation	R_1	$(CH_2O)(NH_4^+)_{1/10} + O_2 \rightarrow HCO_3^- + H^+ + 1/10\ NH_4^+$	$\frac{CTOC * CO2}{\mathrm{CO2} + \mathrm{h1}}$	$\frac{\mathrm{mmolC}}{\mathrm{1(dry\ sediment)y}}$
Heterotrophic denitrification	R_2	$(CH_2O)(NH_4^+)_{1/10} + NO_3^- \rightarrow 2/5\ N_2 + CO_2 + 1/10\ NH_4^+ + 7/5\ H_2O$	$\frac{CTOC * CO2 * CNO3}{(CO2 + h1) * (\mathrm{CNO3} + h2)}$	$\frac{\mathrm{mmolC}}{\mathrm{1(dry\ sediment)y}}$
Manganese oxide reduction	R_3	$(CH_2O)(NH_4^+)_{1/10} + 2\ MnO_2 \rightarrow 1/10\ NH_4^+ + CO_2 + 2\ Mn^{2+} + 3H_2O$	$\frac{CTOC * CO2 * CNO3 * CMnO2}{(CO2 + h1) * (\mathrm{CNO3} + h2) * (CMnO2 + h3)}$	$\frac{\mathrm{mmolC}}{\mathrm{1(dry\ sediment)y}}$
Nitrification	R_4	$NH_4^+ + 2O_2 \rightarrow NO_3^- + H^+ + H_2O$	$k_3 C_{O2} C_{NH4+}$	$\frac{\mu M\ \mathrm{N}}{\mathrm{y}}$
Mn oxidation	R_5	$2\ Mn^{2+} + O_2 + 2\ H_2O \rightarrow 2\ MnO_2 + 4\ H_2O$	$K_4 C_{O2} C_{Mn}^{2+}$	$\frac{\mu M}{\mathrm{y}}$

Table S3. Species and boundary conditions (BC) used in reaction-transport models

Name	Symbol	BC SWI Type (Unit)	BS SWI[a] Value		SBI[b] Values		Reaction Terms ($\sum R_i$)
			3E	4A	3E	4A	
Total organic carbon	CH_2O	Flux (mol m^{-2} yr^{-1})	0.0025	0.0021	--	--	$-R_1-R_2-R_3$
Manganese oxide	MnO_2	Flux (mol m^{-2} yr^{-1})	2E-5	2E-5	--	--	$-R_3$
Oxygen	O_2	Concentration (µM)	250	250	80	190	$-qR_1-2*R_4-R_5$
Ammonium	NH_4^+	Concentration (µM)	0.01	0.01	0.01	0.01	$1/10*q(R_1+R_2+R_3)-R_4$
Nitrate	NO_3^-	Concentration (µM)	21	21	28	23	$-4/5*q*R_2+R_4$
Manganese	Mn^{2+}	Concentration (µM)	0.01	0.01	0.01	0.01	$2*qR_3-2*R_5$

$q = (1-\phi)/\phi$, ϕ = porosity
[a]SWI: sediment-water interface
[b]SBI: sediment-basement interface

Table S4. Reaction-transport parameter values

Name	Symbol	Unit	3E	4A
Solid burial velocity at sediment surface	ω	cm ky-1	0.94	2.00
TOC apparent order of reaction	σ	-	0.15	0.1
Initial distribution of TOC reactivities	α	yr^{-1}	3000	200
R_4 rate constant	k_3	μM^{-1} yr^{-1}	15	1.5
R_5 rate constant	k_4	μM^{-1} yr^{-1}	20	20
Bioturbation coefficient	$D_{b,0}$	cm yr^{-1}	0	0
Biomixing half depth	z_{mix}	cm	7	7
Biomixing attenuation	Z_{att}	cm	0.1	0.1
R_1 O_2 inhibition concentration	h_1	μM	0.5	0.5
R_2 NO_3^- situation concentration	h_2	μM	50	300
R_3 Mn^{2+} situation concentration	H_3	mM	30	30
Bioirrigation coeffcient	α_0	yr^{-1}	0	0
Porosity at sediment surface	φ_0	-	0.70	0.75
Porosity at great depth	φ_∞	-	0.64	0.62
Porosity attenuation coefficient	α_0	cm^{-1}	0.0003	0.0012

Table S5. Primers and thermal conditions

Target genes	Primers	Sequence (5'- 3')	PCR conditions	qPCR	Ref.
Bacterial SSU rRNA gene	Bac341f<br>Uni518r	CCTACGGGWGGCWGCA<br>ATTACCGCGGCTGCTGG	95°C for 15 min, 40× (95°C for 15 s, 58°C for 30 s, 72°C for 30 s)	+	19
Archaeal SSU rRNA gene	Uni515F<br>Arc908r	CAGCMGCCGCGGTAA<br>CCCGCCAATTCCTTTAAGTT	95°C for 15 min, 40× (95°C for 15 s, 60°C for 30 s, 72°C for 45 s)	+	20
Bacteroial *amoA*	AmoA1F<br>AmoA2R	GGGGTTTCTACTGGTGGT<br>CCCCTCKGSAAAGCCTTCTTC	95°C for 15 min, 45× (94°C for 15 s, 55°C for 45 s, 72°C for 60 s)	+	21
Archaeal *amoA*	CrenamoA23f<br>CrenamoA616r	ATGGTCTGGCTWAGACG<br>GCCATCCATCTGTATGTCCA	95°C for 15 min, 40× (95°C for 30 s, 50°C for 45 s, 72°C for 45 s)	+	22
Nitrite oxidizer *nxrB*	nxrB169f<br>nxrB638r	TACATGTGGTGGAACA<br>CGGTTCTGGTCRATCA	95°C for 15 min, 45× (95°C for 15 s, 56.2°C for 40 s, 72°C for 90 s)	+	23
Denitrifier *nirS*	nirS_cd3aF<br>nirS_R3cd	GTSAACGTSAAGGARACSGG<br>GASTTCGGRTGSGTCTTGA	95°C for 15 min, 45× (95°C for 15 s, 51°C for 30 s, 72°C for 45 s)	+	24
Denitrifier *nirK*	nirK_F1aCu<br>nirK_R3Cu	ATCATGGTSCTGCCGCG<br>GCCTCGATCAGRTTGTGGTT	95°C for 15 min, 45× (95°C for 30 s, 56°C for 45 s, 72°C for 45 s, 80°C for 20 s)	+	24
Nitrate-reducer *narG*	narG1960F<br>narG2650R	TAYGTSGGSCARGARAA<br>TTYTCRTACCABGTBGC	95°C for 5 min, 8× (94°C for 30 s, 59°C (-0.5°C/cycle) for 30 s, 72°C for 45 s), 32 ×(94°C for 30 s, 55°C for 30 s, 72°C for 45 s), 72°C for 10 min	+	25
Nitrate-reducer *napA*	napA V67_F<br>napA V67_R	TAYTTYYTNHSNAARATHATG TAYGG<br>DATNGGRTGCATYTCNGCCAT RTT	95°C for 5 min, 40× (94°C for 45 s, 50°C for 45 s, 72°C for 60 s), 72°C for 10 min.	-	26
Denitrifier *nosZ*	nosZ-F<br>nosZ-R	CGYTGTTCMTCGACAGCCAG<br>CATGTGCAGNGCRTGGCAGAA	95 for 5 min, 1× (94°C for 20 s, 65°C for 30 s, 72°C for 40 s), 2× (94°C for 20 s, 62°C for 30 s, 72°C for 40 s), 3× (94°C for 20 s, 59°C for 30 s, 72°C for 40 s), 5× (94°C for 20 s, 57°C for 30 s, 72°C for 40 s), 24× (94°C for 20 s, 55°C for 30 s, 72°C for 40 s), 72 for 10 min.	-	27
Sulfate reducer *dsrB*	DSRp2060F<br>DSR4R	CAACATCGTYCAYACCCAGGG<br>GTGTAGCAGTTACCGCA	95°C for 15 min, 35× (95°C for 35 s, 54°C for 35 s, 72°C for 30 s, 75°C for 10 s)	-	28<br>29
Anammox *hzsA*	hzsA_526F<br>hzsA_1857R	TAYTTTGAAGGDGACTGG<br>AAABGGYGAATCATARTGGC	96°C for 15 min, 40× (96°C for 30 s, 55°C for 30s, 72°C for 30 s)	-	30
DNRA (*nrfA*)	nrfAF2aw<br>nrfAR1	CARTGYCAYGTBGARTA<br>TWNGGCATRTGRCARTC	95°C for 15 min, 35 × (95°C for 30 s, 53°C for 30 s, 72°C for 40 s)	-	31
Nitrogen fixation (*nifH*)	nifHfw<br>nifHrv	GGHAARGGHGGHATHGGNAA RTC<br>GGCATNGCRAANCCVCCRCAN AC	94°C for 15 min, 40 × (94°C for 30 s, 55°C for 30 s, 72°C for 1 min)	-	32

Table 6a. Percentages of various functional groups in the amplicon library from site 3E

Depth	Ammonia oxidizers			Nitrite oxidizers		Denitrifiers			
	Nitroso pumilales	*Nitrosomonas*	*Nitrosococcus*	*Nitrospira*	*Nitrospina*	*Arcobacter*	*Woeseiaceae*	*Pseudomonas*	*Aeromonas*
1H-1	21.2%	0.3%	2.5%	2.4%	4.8%	--	7.8%	--	--
2H-1	11.2%	0.1%	--	0.2%	8.8%	--	0.8%	--	0.04%
2H-2	11.1%	0.1%	0.6%	1.0%	5.4%	0.2%	2.2%	0.1%	--
2H-4	7.5%	0.2%	0.1%	--	6.2%	1.2%	1.9%	0.2%	0.14%
2H-6	6.6%	0.2%	--	0.1%	--	0.8%	1.1%	0.3%	--
3H-3	2.4%	0.3%	--	0.1%	2.5%	0.2%	2.1%	1.4%	--
3H-6	5.9%	0.5%	0.1%	0.4%	2.5%	0.1%	--	1.1%	0.04%
4H-2	8.9%	0.2%	0.4%	1.9%	3.3%	0.2%	2.4%	0.1%	--
4H-3	6.0%	0.1%	0.2%	0.5%	1.1%	--	2.1%	0.3%	0.05%
4H-4	4.9%	--	--	--	--	--	--	0.2%	--
4H-5	0.4%	0.2%	--	--	--	0.4%	--	0.1%	0.01%
4H-6	2.5%	0.1%	--	--	1.7%	--	--	0.1%	0.27%
4H-7	2.4%	0.1%	--	--	0.1%	0.1%	0.2%	0.3%	--
5H-2	1.0%	0.1%	--	--	1.7%	1.1%	--	0.1%	--
5H-4	1.5%	0.1%	--	0.2%	0.7%	0.8%	--	1.1%	--
5H-6	2.6%	0.4%	--	--	1.4%	0.1%	--	0.5%	--
6H-2	2.2%	0.1%	--	--	0.7%	0.1%	--	0.1%	--
6H-5	1.0%	0.3%	--	--	0.3%	0.5%	0.1%	--	--
6H-6	0.9%	0.1%	--	--	0.4%	0.6%	0.2%	1.4%	--

Table S6b. Percentages of various functional groups in the amplicon library from site 4A

Depth	Ammonia oxidizers			Nitrite oxidizers		Denitrifiers			
	Nitroso pumilales	*Nitrosomonas*	*Nitrosococcus*	*Nitrospira*	*Nitrospina*	*Arcobacter*	*Woeseiacea*	*Pseudomonas*	*Aeromonas*
1H-1	67.9%	0.1%	1.8%	0.8%	1.9%	--	4.7%	--	--
1H-2	50.7%	--	0.1%	0.1%	3.1%	--	1.7%	0.3%	--
1H-3	32.0%	0.6%	--	--	2.7%	--	0.9%	0.6%	--
2H-2	41.7%	--	--	0.1%	1.4%	--	2.3%	0.8%	--
2H-6	--	--	--	--	--	--	--	--	--
3H-3	20.8%	--	--	--	2.0%	--	--	3.3%	--
3H-6	23.0%	--	--	--	--	--	--	--	--
4H-2	13.9%	--	--	--	--	--	--	5.4%	--
4H-4	4.0%	--	--	--	4.2%	--	3.0%	0.02%	--
4H-6	53.2%	--	0.9%	1.1%	1.0%	--	4.0%	0.3%	--
5H-3	7.0%	--	--	--	--	--	--	3.6%	--
5H-5	--	--	--	--	1.3%	--	--	--	1.2%
5H-6	--	--	--	--	0.02%	--	--	2.4%	--
6H-3	7.6%	--	--	--	--	--	--	1.6%	--
6H-6	15.6%	--	--	--	1.4%	--	--	3.0%	--
7H-2	33.3%	--	--	--	0.6%	--	--	0.8%	--
7H-6	8.5%	--	--	--	1.6%	--	--	0.7%	--
8H-3	3.2%	--	--	--	1.8%	--	--	0.2%	0.2%
8H-6	6.9%	--	--	--	--	--	--	2.9%	0.1%
9H-3	1.0%	--	--	--	--	--	--	8.9%	--
9H-5	0.1%	--	--	--	--	--	--	1.9%	--
10H-3	39.1%	--	--	--	--	--	--	0.9%	--
10H-5	--	--	--	--	--	--	--	3.9%	--
11H-2	--	--	--	--	0.01%	--	--	18.2%	--

Supplementary References

1 Soetaert, K., Petzoldt, T. & Meysman, F. (R package version, 2010).

2 Treusch, A. H. *et al.* Novel genes for nitrite reductase and Amo-related proteins indicate a role of uncultivated mesophilic crenarchaeota in nitrogen cycling. *Environmental Microbiology* **7**, 1985-1995 (2005).

3 Hamady, M., Walker, J. J., Harris, J. K., Gold, N. J. & Knight, R. Error-correcting barcoded primers allow hundreds of samples to be pyrosequenced in multiplex. *Nature methods* **5**, 235 (2008).

4 Edgar, R. C. UPARSE: highly accurate OTU sequences from microbial amplicon reads. *Nature methods* **10**, 996-998 (2013).

5 Edgar, R. C. Search and clustering orders of magnitude faster than BLAST. *Bioinformatics* **26**, 2460-2461 (2010).

6 Jørgensen, S. L. & Zhao, R. Microbial inventory of deeply buried oceanic crust from a young ridge flank. *Frontiers in Microbiology* **7**, 820 (2016).

7 Lanzen, A. *et al.* CREST - Classification resources for environmental sequence tags. *PloS One* **7**, e49334 (2012).

8 Oksanen, J. *et al.* The vegan package. *Community ecology package* **10**, 631-637 (2007).

9 Galili, T. dendextend: an R package for visualizing, adjusting and comparing trees of hierarchical clustering. *Bioinformatics* **31**, 3718-3720 (2015).

10 Katoh, K. & Standley, D. M. MAFFT Multiple Sequence Alignment Software Version 7: Improvements in Performance and Usability. *Molecular Biology and Evolution* **30**, 772-780, (2013).

11 Stamatakis, A. RAxML-VI-HPC: maximum likelihood-based phylogenetic analyses with thousands of taxa and mixed models. *Bioinformatics* **22**, 2688-2690 (2006).

12 Boudreau, B. P. & Ruddick, B. R. On a reactive continuum representation of organic matter diagenesis. *American Journal of Science* **291**, 507-538 (1991).

13 Ziebis, W. *et al.* Interstitial fluid chemistry of sediments underlying the North Atlantic gyre and the influence of subsurface fluid flow. *Earth and Planetary Science Letters* **323**, 79-91 (2012).

14 Expedition;336;Scientists. Sediment and basement contact coring. *In Edwards, K.J., Bach, W., Klaus, A., and the Expedition 336 Scientists, Proc. IODP, 336: Tokyo (Integrated Ocean Drilling Program Management International, Inc.).* , (2012).

15 Jørgensen, B. B. & Marshall, I. P. G. in *Annual Review of Marine Science* Vol. 8 (eds C. A. Carlson & S. J. Giovannoni) 311-332 (2016).

16 D'Hondt, S. *et al.* Subseafloor sedimentary life in the South Pacific Gyre. *Proceedings of the National Academy of Sciences of the United States of America* **106**, 11651-11656 (2009).

17 Billen, G. Evaluation of nitrifying activity in sediments by dark 14C-bicarbonate incorporation. *Water Research* **10**, 51-57 (1976).

18 Wuchter, C. *et al.* Archaeal nitrification in the ocean. *Proceedings of the National Academy of Sciences of the United States of America* **103**, 12317-12322 (2006).

19 Jørgensen, S. L. *et al.* Correlating microbial community profiles with geochemical data in highly stratified sediments from the Arctic Mid-Ocean Ridge. *Proceedings of the National Academy of Sciences of the United States of America* **109**, 2846-2855 (2012).

20 Ovreås, L., Forney, L., Daae, F. L. & Torsvik, V. Distribution of bacterioplankton in meromictic Lake Saelenvannet, as determined by denaturing gradient gel electrophoresis of PCR-amplified gene fragments coding for 16S rRNA. *Appl. Environ. Microbiol.* **63**, 3367-3373 (1997).

21 Rotthauwe, J. H., Witzel, K. P. & Liesack, W. The ammonia monooxygenase structural gene amoA as a functional marker: Molecular fine-scale analysis of natural ammonia-oxidizing populations. *Appl. Environ. Microbiol.* **63**, 4704-4712 (1997).

22 Tourna, M., Freitag, T. E., Nicol, G. W. & Prosser, J. I. Growth, activity and temperature responses of ammonia-oxidizing archaea and bacteria in soil microcosms. *Environmental Microbiology* **10**, 1357-1364 (2008).

23 Pester, M. *et al.* NxrB encoding the beta subunit of nitrite oxidoreductase as functional and phylogenetic marker for nitrite - oxidizing Nitrospira. *Environmental microbiology* **16**, 3055-3071 (2014).

24 Throback, I. N., Enwall, K., Jarvis, A. & Hallin, S. Reassessing PCR primers targeting nirS, nirK and nosZ genes for community surveys of denitrifying bacteria with DGGE. *FEMS Microbiol. Ecol.* **49**, 401-417 (2004).

25 Philippot, L., Piutti, S., Martin-Laurent, F., Hallet, S. & Germon, J. C. Molecular analysis of the nitrate-reducing community from unplanted and maize-planted soils. *Appl. Environ. Microbiol.* **68**, 6121-6128 (2002).

26 Lam, P. *et al.* Revising the nitrogen cycle in the Peruvian oxygen minimum zone. *Proceedings of the National Academy of Sciences of the United States of America* **106**, 4752-4757 (2009).

27 Kloos, K., Mergel, A., Rosch, C. & Bothe, H. Denitrification within the genus Azospirillum and other associative bacteria. *Australian Journal of Plant Physiology* **28**, 991-998 (2001).

28 Wagner, M., Roger, A. J., Flax, J. L., Brusseau, G. A. & Stahl, D. A. Phylogeny of dissimilatory sulfite reductases supports an early origin of sulfate respiration. *Journal of Bacteriology* **180**, 2975-2982 (1998).

29 Geets, J. *et al.* DsrB gene-based DGGE for community and diversity surveys of sulfate-reducing bacteria. *Journal of Microbiological Methods* **66**, 194-205 (2006).

30 Harhangi, H. R. *et al.* Hydrazine synthase, a unique phylomarker with which to study the presence and biodiversity of anammox bacteria. *Appl. Environ. Microbiol.* **78**, 752-758 (2012).

31 Welsh, A., Chee-Sanford, J. C., Connor, L. M., Loeffler, F. E. & Sanford, R. A. Refined NrfA phylogeny improves PCR-based nrfA gene detection. *Appl. Environ. Microbiol.* **80**, 2110-2119 (2014).

32 Mehta, M. P., Butterfield, D. A. & Baross, J. A. Phylogenetic diversity of nitrogenase (nifH) genes in deep-sea and hydrothermal vent environments of the Juan de Fuca ridge. *Appl. Environ. Microbiol.* **69**, 960-970 (2003).
